# Amino acid analysis for peptide quantitation using reversed-phase liquid chromatography combined with multiple reaction monitoring mass spectrometry

**DOI:** 10.1007/s00216-023-04840-2

**Published:** 2023-07-19

**Authors:** Deema O. Qasrawi, Evgeniy V. Petrotchenko, Christoph H. Borchers

**Affiliations:** 1 Segal Cancer Proteomics Centre, Lady Davis Institute for Medical Research, Jewish General Hospital, McGill University, Montreal, Quebec Canada; 2 Gerald Bronfman Department of Oncology, McGill University, Montreal, Quebec Canada; 3 Segal Cancer Centre, Lady Davis Institute for Medical Research, Jewish General Hospital, McGill University, Montreal, Quebec Canada; 4 Department of Pathology, McGill University, Montreal, Quebec Canada; 5 Division of Experimental Medicine, McGill University, Montreal, Quebec Canada

**Keywords:** LC-MRM-MS, Hydrochloric acid hydrolysis, Amino acid analysis (AAA), Peptide quantification, Stable isotope-labeled internal standards

## Abstract

Amino acid analysis (AAA) can be used for absolute quantitation of standard peptides after acid hydrolysis using 6 M HCl. Obtained individual amino acids can then be quantified by liquid chromatography-mass spectrometry (LC–MS). Achieving baseline separation of non-derivatized amino acids is challenging when reversed-phase (RP) chromatography is used. Several derivatization methods are commonly utilized to address this issue; however, derivatization has several drawbacks, such as derivative instability and lack of reproducibility. Currently, separation of non-derivatized amino acids is typically done using HILIC, but HILIC has problems of poor reproducibility and long column equilibration times. We developed a method to quantify non-derivatized amino acids, including methionine and cysteine, from peptide hydrolysates by RP-LC-MS without special pre-treatment of the samples. Samples were spiked with certified isotopically labeled (^13^C- and/or ^15^N-) amino acids as internal standards. The amino acids released from acid hydrolysis were then analyzed by RP-UPLC-MRM-MS and quantified using the analyte/internal standard chromatographic peak area ratios. Peptide quantitation was based on the sum of the individual amino acid concentrations from the known peptide sequences. The resulting method did not require derivatization, used standard C18-based reversed-phase liquid chromatography, did not require external calibration, was robust, and was able to quantify all 17 amino acids for which we had internal standards, including the sulfur-containing amino acids, cysteine and methionine, in their respective oxidized forms. This simple and robust method enabled the absolute quantitation of standard peptides using only acid hydrolysis and a standard RP-UPLC-MRM-MS setup.

**Graphical Abstract:**

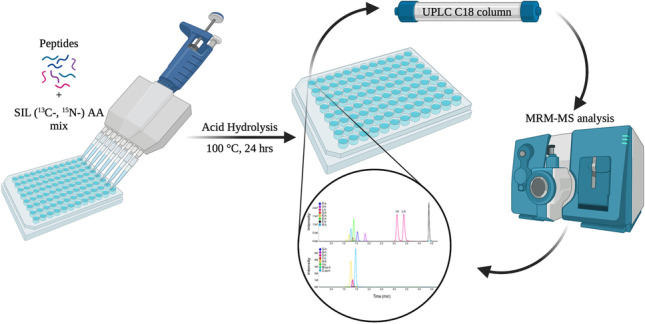

**Supplementary Information:**

The online version contains supplementary material available at 10.1007/s00216-023-04840-2.

## Introduction

Peptides and proteins are biomolecules consisting of amino acids bound together covalently via peptide bonds. Amino acid analysis (AAA) determines the amino acid content of proteins or peptides by hydrolyzing the peptide bonds to generate free amino acids [1]. Moore and Stein introduced the acid hydrolysis method in the early 1950s [2–4]. This standard classical hydrolysis method uses 6 M HCl and heating to 110 °C for 18–24 h [5, 6]. Several earlier analytical methods used gas chromatography (GC) or high-performance liquid chromatography (HPLC) to separate and analyze free amino acids, but achieving baseline separation of underivatized amino acids on reversed-phase (RP) chromatography is challenging due to the polar nature of free amino acids. Thus, methods using derivatization have dominated amino acid separation and detection by liquid chromatography [7] as it enables baseline separation of the amino acids by RP-HPLC. Since most amino acids lack chromophores, a derivatization step is also necessary to make the compounds detectable by UV or fluorescence [8]. Most derivatization techniques, however, have drawbacks, such as derivative instability, poor reproducibility in derivatization yield, reagent interferences, and/or lengthy and tedious derivatization protocols [9]. Liquid chromatography combined with mass spectrometry (LC-MS) has high selectivity and sensitivity for detecting both derivatized and underivatized amino acids [10–12] without requiring derivatization. Hydrophilic interaction liquid chromatography coupled with mass spectrometry (HILIC-LC-MS) is the current “gold standard” chromatographic technique for analyzing underivatized amino acids [9, 13, 14]. However, it has several drawbacks and limitations, including poor reproducibility and a long column equilibration time compared to the RP-LC-MS [8, 9, 15].

The goal of our current study was to develop a RP-UPLC-MRM-MS method for the quantitation of underivatized amino acids from peptide hydrolysates, using simple sample preparation and robust chromatography, and to use this method in a high-throughput format for routine analysis.

## Experimental

### Reagents

A kit containing 21 L-amino acid standards, a mixture of 17 certified stable isotope-labeled amino acids at a concentration of 2.5 mMl/L each (in 0.1 N HCl), human angiotensin II (DRVYIHPF), HPLC-MS/MS grade acetonitrile, water, formic acid, and 6 N hydrochloric acid solution were purchased from Sigma-Aldrich (St. Louis, MO, USA). Glass screw-cap vials (PN C4013-492A) were purchased from Thermo (Waltham, MA, USA). Hybridization bags Hybri-Bag Hard (PN SE-S-1001-EX) were purchased from Cedarlane (Burlington, ON, CA).

### Experimental procedures

#### Standard preparation

The working stock solution of isotopically labeled amino acid internal standards was prepared by diluting the original commercial 2.5 mM solution of certified isotopically labeled amino acid mixture with 0.1% aqueous formic acid to generate a concentration of 0.25 mM of each amino acid. The prepared working stock solution was aliquoted and stored at − 20 °C until analysis. The working stock solution was diluted in 0.1% aqueous formic acid to obtain a working solution of 30 µM of each amino acid. Non-labeled amino acid standards were prepared as 10 mg/mL stock solutions in either water or 1 M HCl, according to the manufacturer’s recommendations. All of the prepared solutions were aliquoted, stored at − 20 °C and brought to room temperature before use.

#### Quality-control material

Angiotensin II (ANGII) (sequence: DRVYIHPF, molecular weight: 1046.18, peptide content: 72.6%) was used as a quality-control material. The actual ANGII concentration was adjusted based on the peptide content. A stock solution at 1 mM was prepared by reconstituting the content of the vial with 0.1% formic acid in water. The stock solution was aliquoted, stored at -20 °C, and brought to room temperature before use.

#### Sample preparation

Acid hydrolysis of the peptide samples and all of the sample preparation steps were performed in autosampler vials with glass inserts, which streamlined sample processing and analysis. A 10-µL peptide sample at an expected theoretical concentration of 30–50 µM was combined with 10 µL of isotopically labeled amino acid internal standards of isotopically labeled amino acid internal standards, dried in vacuo using a SpeedVac (Labconco), reconstituted by adding 50 µL of 6 M HCl, and sealed with heat-resistant autosampler vial caps with PTFE septa. The hydrolysis was performed at 120 °C for 24 h in a dry heater block. Vials were cooled at room temperature, caps were loosened, and the hydrolysates were dried in vacuo*.* Samples were reconstituted by adding 30 µL of 0.1% aqueous formic acid. One microliter (equivalent to approximately 10 pmol of each peptide) was used for the analysis.

For the high-throughput amino acid analysis, the acid hydrolysis and sample preparation steps were performed in a polypropylene 96-well PCR plate (Eppendorf or Nest Biotechnology). The sample in each well was prepared as described above for the vial inserts. A PCR plate sealer (Bio-Rad) was used to seal the plate with the hybridization bag film at 180 °C for 6 s. The hydrolysis was performed using the C1000 Touch PCR thermocycler (Bio-Rad) at 100 °C for 24 h. Before drying down the hydrolysates in vacuo, the plate was cooled at room temperature and centrifuged, and the seal for each well was cut or punctured. Alternatively, PCR plates were sealed with silicone mats (Axygen), clamped between two metal plates, incubated at 120 °C in the oven (Thermo), and processed in 96-well plates as described above.

#### LC-MRM-MS analysis

An RP-UPLC-MRM-MS analysis was performed using a Nexera XR UPLC system (Shimadzu, Japan) coupled to a Sciex QTrap 6500 + mass spectrometer equipped with an IonDrive Turbo V electrospray ionization source. Chromatographic separation was performed using a Zorbax Eclipse Plus C18 column (2.1 × 150 mm, 1.8 μm, Agilent, USA) with a 3-min gradient (mobile phase A: 0.1% formic acid in HPLC-grade water; mobile phase B: 0.1% formic acid in acetonitrile) at a flow rate of 0.250 mL min^−1^. The LC gradient program started at 0% solvent B and was ramped to 50% in 3 min. Between 3.0 and 3.1 min, the % B was returned to the initial conditions (0%) and held until 6.0 min. The sample injection volume was 1 µL, the column oven temperature was set to 35 °C, and the autosampler temperature was set to 15 °C.

Analysis was performed using electrospray ionization in the positive mode. The MS parameters were as follows: capillary voltage 5.5 kV; source temperature 550 °C; ion source gas 1 at 50 psi; ion source gas 2 at 60 psi. The collision gas was set to medium, and the curtain gas was set to 35 psi. Data acquisition was performed using Analyst 1.6.3 software (Sciex), using multiple reaction monitoring (MRM) with a 10-ms dwell time for each transition. The MRM transitions and the manually optimized acquisition parameters are listed in Table 1.

**Table 1:** Multiple reaction monitoring parameters for amino acids and their respective internal standards on QTRAP 6500 + RP-UPLC-MRM-MS

Amino acid	Precursor ion	Production	CE	EP	DP	CXP
Glycine (Gly, G)	76.1	30.1	20	10	6	14
^13^C_2_, ^15^N-Glycine	79.1	32.1	20	10	6	14
Alanine (Ala, A)	90.1	44.1	16	10	6	6
^13^C_3_, ^15^N-Alanine	94.1	47.1	16	10	6	6
Serine (Ser, S)	106.1	60.1	15	10	6	7
^13^C_3_, ^15^N- Serine	110.1	63.1	15	10	6	7
Proline (Pro, P)	116.1	70.1	21	13	20	10
^13^C_5_-Proline	121.1	74.1	21	13	20	10
Valine (Val, V)	118.1	72.1	27	13	11	8
^13^C_5_-Valine	123.1	76.1	27	13	11	8
Threonine (Thr, T)	120.1	74.1	25	14	105	7
^13^C_4_-Threonine	124.1	77.1	25	14	105	7
Leucine/Isoleucine (Leu, L/Ile, I)	132.1	86.1	13	14	8	10
^13^C_6_, ^15^N- Leucine/Isoleucine	139.1	92.1	13	14	8	10
Aspartic acid (Asp, D)	134.1	74.1	19	14	7	10
^13^C_4_-Aspartic acid	138.1	76.1	19	14	7	10
Lysine (Lys, K)	147.1	84.1	23	13	15	10
^13^C_6_-Lysine	153.1	89.1	23	13	15	10
Glutamic acid (Glu, E)	148.1	84.1	21	14	21	10
^13^C_5_-Glutamic acid	153.1	88	21	14	21	10
Methionine (Met, M)	150.1	104.1	15	12	6	11
^13^C_5_, ^15^N-Methionine	156.1	109.1	15	12	6	11
Histidine (His, H)	156.1	110.1	19	13	16	12
^13^C_6_-Histidine	162.1	115.1	19	13	16	12
Phenylalanine (Phe, F)	166.1	103	37	14	11	12
Phenyl-^13^C_6_-alanine	172.1	109	37	14	11	12
Arginine (Arg, R)	175.1	70.1	27	11	40	8
^13^C_6_-Arginine	181.1	74.1	27	11	40	8
Tyrosine (Tyr, Y)	182.1	136.1	13	11	20	8
Tyrosine-(phenyl-^13^C_6_)	188.1	142.1	13	11	20	8
Cystine	241.2	152.1	20	14	20	10
^13^C_6_, ^15^N_2_-Cystine	249.2	156.1	20	14	20	10
Lysine (+ 8)	155.1	90.1	23	13	15	10
Arginine (+ 10)	185.1	75.1	27	11	40	8
Methionine-Ox	166.1	56.1	15	12	6	11
^13^C_5_, ^15^N-Methionine-Ox	172.1	60.1	15	12	6	11
Cysteine-Ox	170.1	124.1	20	14	20	10
^13^C_6_, ^15^N_2_-Cysteine-Ox	174.1	127.1	20	14	20	10

*CE* collision energy, *EP* entrance potential, *DP* declustering potential, *CXP* collision cell exit potential

The concentration of each amino acid that was present in the peptide sequence was calculated from the chromatographic peak area ratio of the amino acid analyte to the corresponding stable isotopically labeled amino acid internal standard with a known concentration. Glutamine and asparagine were measured in their corresponding hydrolyzed forms, as glutamic acid and aspartic acid, respectively. The concentrations of repeating amino acid residues in the peptide sequence were calculated by dividing the determined amino acid concentrations by the number of repeats in the peptide sequence. The concentration of the peptide was calculated as the average of the concentrations of the individual amino acids present in the peptide sequence. The %CV of each individual amino acid concentration was also calculated and reported.

#### Method evaluation

Hydrolysis time was optimized using a peptide (ILLLIPK) at 5 different time points (1, 18, 20, 24, and 48 h). Three replicates of each independent experiment were used. The RP-UPLC-MRM-MS AAA method was evaluated for intra-day (*n* = 10), and inter-day (*n* = 12) precision using three different peptides: TIIYWDSQTTIEK, TPETVPQVTSK, and ILLLIPK.

Method linearity was evaluated by serial dilution of the highest concentration of the amino acid calibration standard to produce concentrations of 5, 7.5, 12.5, 25, 50, and 100 μM in 0.1% aqueous formic acid spiked with the ^13^C and/or ^15^N-labeled internal standard mixture. Solutions were prepared in triplicate and analyzed by RP-UPLC-MRM-MS. Chromatographic peak area ratios (analyte vs. internal standard) were plotted versus the concentrations of the amino acids, and the coefficient of determination (*r*^2^) was determined. Linearity was further evaluated by serial dilution (0.15–165.5 μM) of the peptide quality control material, ANGII, spiked with ^13^C and/or ^15^N-labeled amino acid internal-standard mixture. The coefficient of determination (*r*^2^) was evaluated for each amino acid present in the peptide sequence using 5 independent replicates.

Analysis accuracy was determined by comparing the ANGII quantitative results to the theoretical values using 10 independent replicates.

## Results and discussion

Knowing the exact concentration of the peptide standard is a prerequisite for quantitative proteomics applications. Absolute quantitation of peptides can be done by hydrolyzing peptides to the constituent amino acids, followed by AAA to determine the absolute amounts of the obtained amino acids. Traditionally, AAA has been performed using derivatization with chromophores or fluorophores, and subsequent HPLC separation. LC–MS, however, can analyze amino acids without the need for derivatization. Due to the polar nature of free amino acids, HILIC chromatography is usually utilized for such analysis, although it has several drawbacks, such as irreproducible retention times, long equilibration times, and sensitivity to variations in the mobile phase composition.

Here, we report the use of standard C_18_-based reversed-phase chromatography, in combination with MRM-MS and the certified isotopically labeled amino-acid standards (for which accurate amino acid concentrations are guaranteed), for the absolute quantitation of free amino acids in peptide hydrolysates. Peptide samples were spiked with a known amount of each isotopically labeled certified amino acid mix, and the mixtures were hydrolyzed using standard liquid-phase acid hydrolysis with 6 M HCl at 100–120 °C. The hydrolysates obtained were dried in vacuo and then reconstituted and analyzed by RP-UPLC-MRM-MS using a short 3-min 0.1% FA water-acetonitrile gradient.

**Fig. 1:**
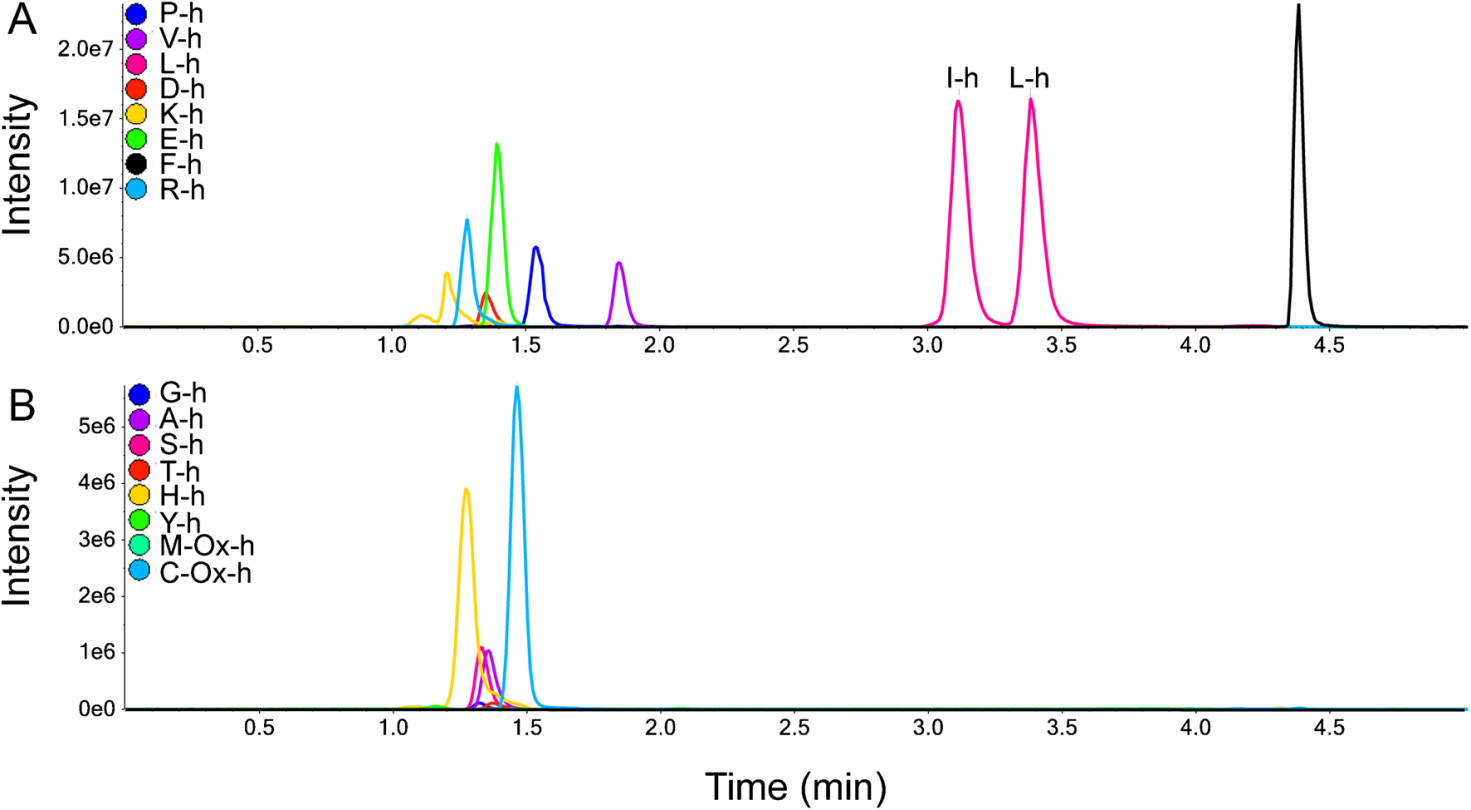
The extracted ion chromatogram (XIC) of the 17 SIL amino acid standard mixture was manually separated into two figures (**A**) and (**B**) to visualize overlapping peaks. The response of the analytes is expressed as ion counts (*y*-axis) vs. time (*x*-axis). The oxidized forms of Met and Cys are designated as M-Ox and C-Ox, respectively. Isotopically labeled internal standards are labeled as -h (heavy)

The method achieved baseline separation of all amino acids with similar precursor ion masses, as well as the isomeric Leu and Ile (Fig. 1). The isotopically labeled amino acids (K + 8, R + 10), which are commonly used for synthesizing isotopically labeled peptides, are different in mass from the certified isotopic standards used for the quantitation and can also be measured (Table 1). Usually, radical scavengers are added to the hydrolysis mixture to prevent oxidation of the sulfur-containing amino acids cysteine and methionine. Here, instead of preventing Cys and Met oxidation, we omitted the use of the scavengers in the hydrolysis reaction and measured Cys and Met in their oxidized forms (cysteic acid and methionine sulfoxide, respectively) [16], relying on the identical oxidation reactions of unlabeled cystine and Met and isotopically labeled cystine and Met standards (cystine converts to cysteic acid upon hydrolysis) (Fig. 2). The same approach could be used for the determination of tryptophan, if the corresponding certified isotopically labeled internal standard would be available.

**Figure:**
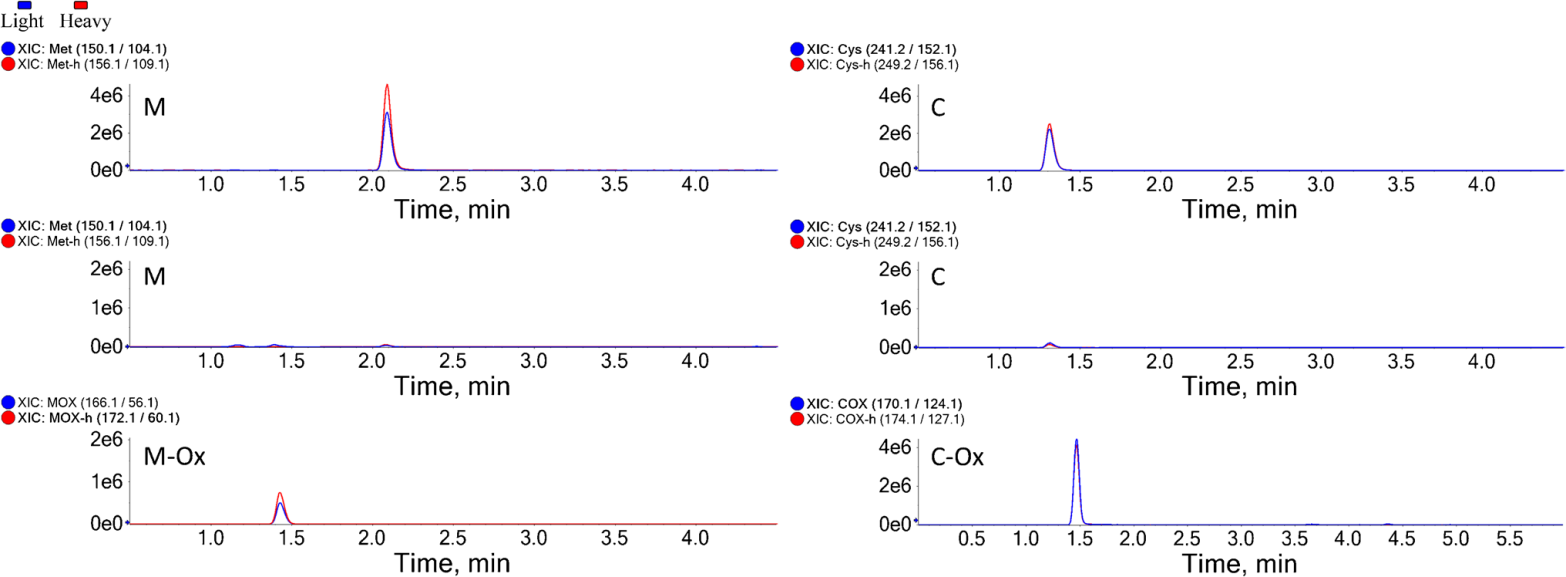
Extracted ion chromatograms (XIC) of detection of the oxidized form of Met (left panel) and Cys (right panel). Both amino acids were detected before acid hydrolysis (top) but were barely detectable after hydrolysis (middle). The oxidized forms of both Met and Cys (designated as M-Ox and C-Ox, respectively) were detectable after hydrolysis (bottom). The response of the analytes is expressed as ion counts (*y*-axis) vs. time (*x*-axis)

Quantitation of the individual amino acids was performed based on chromatographic peak area ratios of the analytes to those of the certified isotopically-labeled amino acid standards. Glutamine and asparagine were measured as their corresponding hydrolyzed forms — i.e., as glutamic acid and aspartic acid, respectively. Thus, this assay actually measures the sum of glutamine plus glutamic acid and the sum of asparagine plus aspartic acid.

Quantitation of the peptides was performed by averaging the values for the content of the amino acids constituting the peptides using a VBScript macro written in-house. The %CV of the determined peptide concentration based on the concentration of each amino acid present in the peptide sequence was calculated and reported. The acceptance %CV threshold value was set at 15%.

The analysis can be performed in a high-throughput format. We perform the hydrolysis and all subsequent sample manipulations in the same glass insert of the autosampler vials, which can then be directly used for LC–MS analysis. This allows the processing 54 samples per 6 × 9 aluminum autosampler vials tray (Thermo) in parallel. In addition, we performed the entire analysis in 96-well polypropylene PCR plates, which can also be used directly for injection into the LC–MS system using an autosampler.

**Fig. 3:**
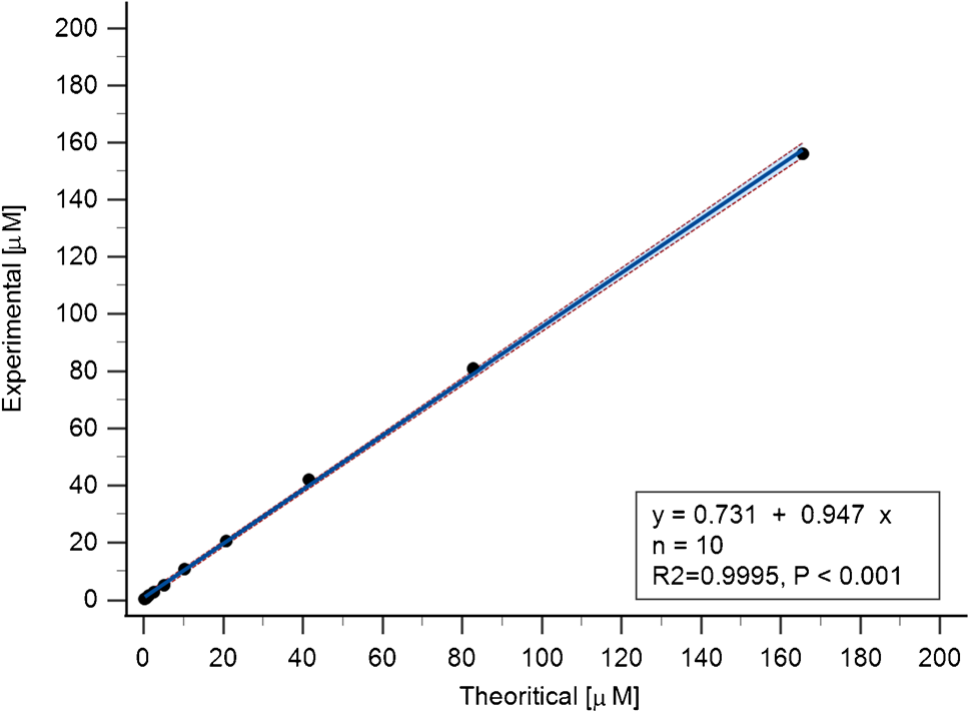
Analysis accuracy. A correlation of the peptide concentration was obtained in AAA for the angiotensin II (*y*-axis) versus the theoretical concentration (*x*-axis). Coefficient of determination (*r*^2^) = 0.9995, *n* = 10 independent experiments

We have confirmed that, under the conditions used, maximum hydrolysis can be achieved in 24 h (Supplementary Figure S1). The method has been evaluated for the linearity of both the amino acid and peptide concentration responses. For each amino acid response, an *r*^2^ of ≥ 0.997 was achieved for the measured interval of 1–100 pmol on column (Supplementary Figure S2). The linearity of the amino acid responses after hydrolysis was evaluated using ANGII QC peptide for each amino acid present in the peptide sequence in 5 independent replicates with a coefficient of determination (*r*^2^) ≥ 0.997 (Supplementary Figure S3). The linearity of the peptide concentration response and the method’s accuracy was assessed by comparing experimentally determined ANGII QC peptide concentrations with theoretical values (Fig. 3). A coefficient of determination (*r*^2^) = 0.9995 and a slope of 0.947 was found (*n* = 10 independent experiments), indicating that the method is accurate. The method precision was found to be satisfactory (intra- and inter-day %CV were ≤ 10%) (Supplementary Table S1), and no carryover was observed.

Overall, a simple and robust method was developed for absolute peptide quantitation utilizing acid hydrolysis and RP-UPLC-MRM-MS analysis with certified isotopically labeled amino-acid internal standards.

## Conclusions

In this study, a new RP-UPLC-MRM-MS method was developed for absolute peptide quantitation using acid hydrolysis and the analysis of underivatized amino acids from peptide hydrolysates with certified isotopically labeled amino acid internal standards. The method does not require the use of radical scavengers, because methionine and cysteine can be quantified in their oxidized forms. The method can be performed in a high-throughput format using either autosampler glass vials with inserts or in 96-well polypropylene PCR plates.

## Supplementary Information

Below is the link to the electronic supplementary material.

Supplementary file1 (DOCX 311 KB)
