# Supplementary material for: Amino acid analysis for peptide quantitation using reversed-phase liquid chromatography combined with multiple reaction monitoring mass spectrometry
Source: Anal Bioanal Chem. 2023 Jul 19;415(22):5261–7. doi: 10.1007/s00216-023-04840-2 (PMC10444640; doi:10.1007/s00216-023-04840-2)
Supplement: Supplementary file 1 — Supplementary file1 (DOCX 311 KB) [file 216_2023_4840_MOESM1_ESM.docx]

**Amino acid analysis for peptide quantitation using reversed-phase liquid chromatography combined with multiple reaction monitoring mass spectrometry**

Deema O. Qasrawi^1^, Evgeniy V. Petrotchenko^1^, Christoph H. Borchers^1,2,3,4,5*^

^1^Segal Cancer Proteomics Centre, Lady Davis Institute for Medical Research, Jewish General Hospital, McGill University, Montreal, Quebec, Canada

^2^Gerald Bronfman Department of Oncology, McGill University, Montreal, Quebec, Canada

^3^Segal Cancer Centre, Lady Davis Institute for Medical Research, Jewish General Hospital, McGill University, Montreal, Quebec, Canada

^4^Department of Pathology, McGill University, Montreal, Quebec, Canada

^5^Division of Experimental Medicine, McGill University, Montreal, Quebec, Canada

***Corresponding author:**

Christoph Borchers

Segal Cancer Proteomics Centre, Lady Davis Institute for Medical Research,

Jewish General Hospital, McGill University,

Montreal, Quebec, Canada

Email address: christoph.borchers@mcgill.ca

**SUPPLEMENTARY MATERIAL**

**
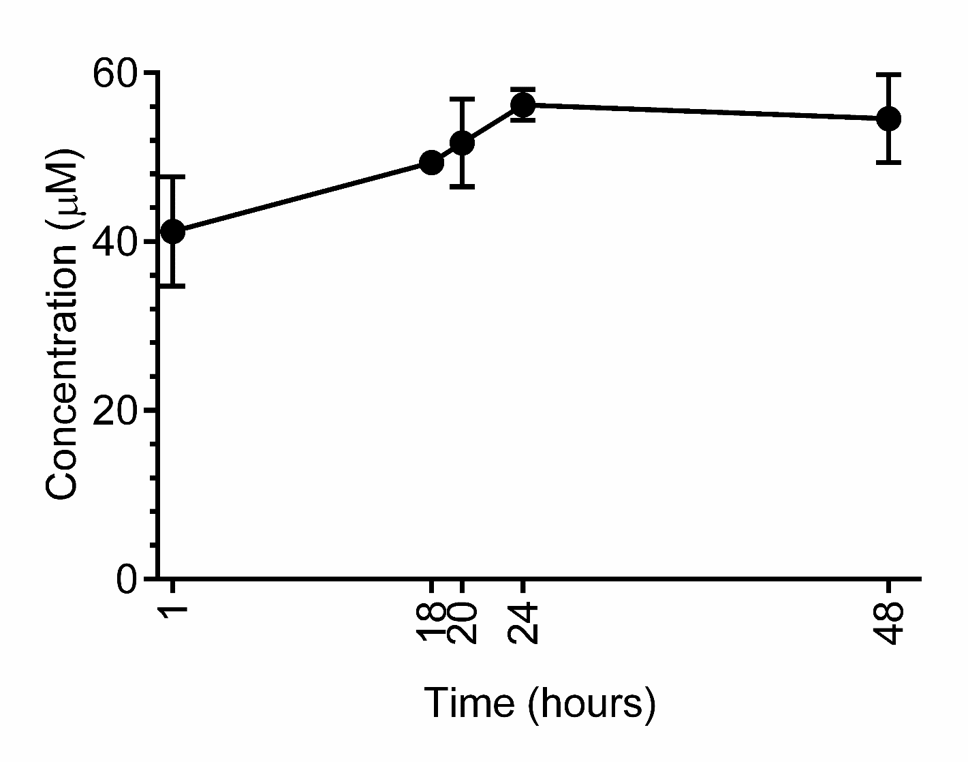
Supplementary Figures**

**SupplementaryFigure S1. Hydrolysis time optimization using a peptide (ILLLIPK) at different time points (1, 18, 20, 24, and 48 hr).** Maximal hydrolysis can be achieved in 24 hours. Values represent means ± S.E.M. for N = 3 independent experiments.

**SupplementaryFigure S2. Linearity for unhydrolyzed amino acid responses.** Standard curves were generated using a serial dilution (5-100 μM), spiked with ^13^C, ^15^N labeled internal standard mixture. Ratios of peak areas (analyte vs internal standard, y-axis) were plotted against concentrations of amino acids (x-axis). Hydrolysis and analysis for each point were carried out using 96 well plates in 5 replicates. Coefficient of determination (r^2^) ≥ 0.997.

|  |
| --- |

**SupplementaryFigure S3. Linearity for amino acid responses in peptide hydrolysate.** Standard curves were generated using a serial dilution (0.15-165.5 μM) of Angiotensin II (sequence: DRVYIHPF), spiked with a ^13^C and/or ^15^N-labeled amino acid internal standard mixture. Ratios of peak areas (analyte vs internal standard) were plotted against concentrations of each amino acid (x-axis). Hydrolysis and analysis for each point were carried out using 96 well plate in 5 replicates. Coefficient of determination (r^2^) ≥ 0.997, n=5 independent experiments.

Supporting Information

**Supplementary Table S1. Intra- and inter-day method variation**

|  | | | Inter-day (between run) | | | | |  | | | | | | \\\ | | | Intra-day (within run) | | |
| --- | --- | --- | --- | --- | --- | --- | --- | --- | --- | --- | --- | --- | --- | --- | --- | --- | --- | --- | --- |
|  | **Peptide 1** | | | **Peptide 2** | | **Peptide 3** | | |  | |  | | **Peptide 1** | | | **Peptide 2** | | | **Peptide 3** |
| **Peptide sequence** | | TIIYWDSQTTIEK | | | TPETVPQVTSK | | ILLLIPK | | | **Peptide sequence** | | TIIYWDSQTTIEK | | | TPETVPQVTSK | | | ILLLIPK | |
| **Expected Conc. [µM]** | | 31 | | | 56 | | 66 | | | **Expected Conc. [µM]** | | 31 | | | 56 | | | 66 | |
| **Replicates** | | 9 | | | 9 | | 9 | | | **Replicates** | | 6 | | | 6 | | | 6 | |
| **Mean [µM]** | | 23.77 | | | 42.95 | | 50.71 | | | **Mean [µM]** | | 22.89 | | | 41.38 | | | 51.3 | |
| **CV%** | | 9.76 | | | 6.16 | | 6.45 | | | **CV%** | | 10.31 | | | 5.53 | | | 1.96 | |
